# Giant Lipoma of the Parapharyngeal Space: Surgical Considerations and Comprehensive Literature Review

**DOI:** 10.1155/crot/5564843

**Published:** 2026-04-29

**Authors:** Amy Wang, Nicholas A. Rossi, Viran Ranasinghe

**Affiliations:** ^1^ Department of Otolaryngology, Vanderbilt University Medical Center, Nashville, Tennessee, USA, vanderbilt.edu; ^2^ Department of Otolaryngology, University of Texas Medical Branch, Galveston, Texas, USA, utmb.edu

**Keywords:** case report, facial nerve dissection, lipoma, neck dissection, otolaryngology surgery, parapharyngeal space

## Abstract

**Background:**

The rarity of pharyngeal space lipomas (PPL) combined with the complex anatomy of the region makes both diagnosis and treatment challenging. We introduce the case of a patient with massive PPL with significant superior extension. Additionally, a review of the literature is presented with the aim of characterizing trends in presentation, diagnostic workup, and common surgical approaches to PPLs.

**Case Presentation:**

A 62‐year‐old male patient presented with a left‐sided neck mass that had been progressively enlarging over several years. CT Soft Tissue Neck with contrast displayed a well‐circumscribed mass measuring a maximum 10.6 cm in dimension in the posterior triangle of the neck, extending from the skull base within the parapharyngeal space to the supraclavicular fossa. The mass was subsequently excised through a transcervical surgical approach with facial nerve dissection, avoiding the need for mandibulotomy. Permanent section pathologic analysis was consistent with a simple lipoma. At the 1‐month postoperative visit, the patient had recovered uneventfully with no evidence of cranial nerve deficits or dysphagia.

**Review of Literature:**

A literature search was conducted on PubMed for “parapharyngeal” + “lipoma”. Since 1977, there have been 41 articles published on PPL with a total of 45 patients. A majority of patients are male (78%). The median maximum dimension of PPLs measured 7.2 cm, larger than other parapharyngeal space tumors. Currently, MRI and CT are the most commonly employed imaging modalities. Transcervical was the most popular surgical approach (*n* = 16, 36%), followed by transparotid (*n* = 7, 16%) and transoral (*n* = 5, 11%).

**Conclusion:**

PPL management demands intricate preoperative planning, particularly when the mass extends into challenging anatomical territories. Advanced preoperative imaging, detailed understanding of anatomy, and a tailored surgical approach are key to management of tumors in the parapharyngeal space.

## 1. Introduction

The parapharyngeal space represents a clinically significant yet anatomically intricate location for head and neck surgeons [1, 2]. While tumors occurring in the parapharyngeal space are rare, accounting for less than 2% of all head and neck tumors [3], these lesions may encase or displace critical anatomical structures such as the internal carotid artery, internal jugular vein, and lower cranial nerves, requiring meticulous surgical planning and execution [4–8]. Lipomatous lesions are an especially uncommon presentation, with reported occurrences ranging from less than 1% to approximately 6% of all parapharyngeal space tumors [9–11]; however, their benign nature should not discount the complexities involved in their surgical management.

We introduce the case of a 62‐year‐old male patient who presented with a massive parapharyngeal space lipoma (PPL) with a maximum dimension of 10.6 cm and significant superior extension, successfully treated with avoidance of mandibulotomy through transcervical excision. Additionally, a review of the literature is presented with the aim of characterizing trends in presentation, diagnostic workup, and common surgical approaches to PPLs. Current literature is limited to case reports, and there is no existing consensus on management and outcomes of these lipomas. We discuss our experience as well as the general operative nuances of PPLs, and a summary of clinical features, diagnostic imaging guidance, and options for surgical approach is provided.

## 2. Case Presentation

A 62‐year‐old male patient presented for evaluation of a progressively enlarging left‐sided neck mass. The history of neck mass was of uncertain duration but had been noticeable for several years and was steadily growing. The patient reported a recent increase in discomfort with full neck range of motion, prompting a desire for removal, but otherwise had no functional or neurologic deficits. The patient’s past medical history was limited to hypercholesterolemia and Type 2 diabetes mellitus which was well‐controlled with oral hypoglycemic agents. He denied weight loss or a history of tobacco use and reported no symptoms of dysphagia or odynophagia, dysphonia, dyspnea, or sleep apnea.

Physical examination revealed a diffusely palpable, nonpulsatile soft left neck mass with limited mobility encompassing the entirety of the left lateral neck from the preauricular region to the supraclavicular space. There was additionally a medial‐based bulge of the left oropharyngeal sidewall and slight gross displacement of oropharyngeal structures, but no other abnormalities were present. Mucosa remained intact. Cranial nerve examination was within normal limits.

**FIGURE 1:**
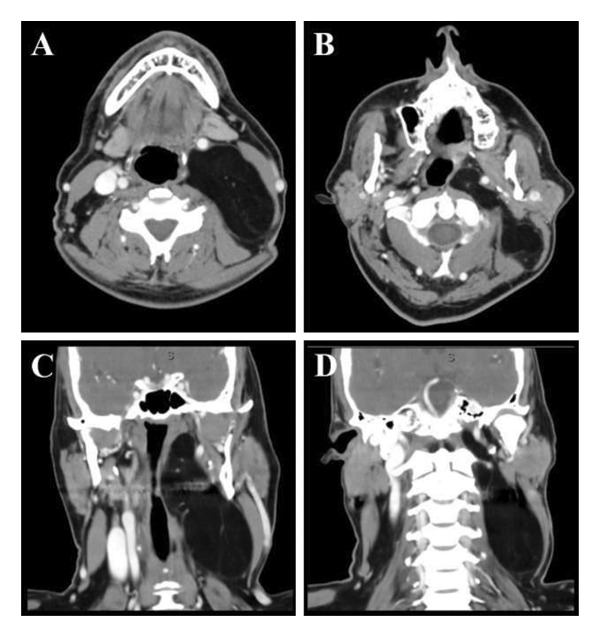
CT neck with contrast (A‐B: axial plane; C‐D: coronal plane): Depicted here is a 10.6 × 6.8 × 3.6 cm well‐circumscribed mass of fat density encompassing the left lateral neck from the supraclavicular fossa up to the skull base. Note the absence of normal internal jugular vein in images (A) and (C). Image (B) depicts the medial extent of the mass into the parapharyngeal space. Image (D) illustrates the superior extent up to the skull base.

CT soft tissue neck with contrast revealed a 10.6 × 6.8 × 3.6 cm well‐circumscribed, minimally attenuating left‐sided poststyloid mass deep to the sternocleidomastoid. The dimension of the mass was greatest lengthwise, and it encompassed the posterior triangle of the neck from the skull base within the parapharyngeal space to the supraclavicular fossa. The central portion of the mass protruded into both the nasopharynx and oropharynx, resulting in medial displacement of the structures. There was considerable displacement anteriorly of the carotid artery as well as compression of the internal jugular vein (Figure 1). While the dimensions were atypically large for a parapharyngeal mass, the appearance of the mass was consistent with a lipomatous lesion without any concurrent soft tissue densities, significant septations, or calcifications. Thus, given the lipomatous presentation and the absence of any signs concerning malignancy, additional imaging with MRI and a potential biopsy were deferred.

There were no perioperative concerns. The patient was given the option to continue observation of the mass or pursue surgical excision, and the decision was made to proceed to the operating room. He was counseled preoperatively on the risk of lower cranial nerve injury and possibility of dysphagia associated with surgery.

### 2.1. Treatment and Outcome

**FIGURE 2:**
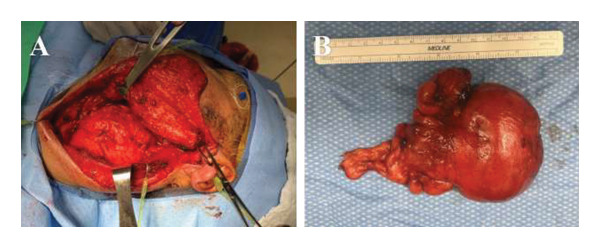
Gross pathological specimen, postexcision: Pictured below are gross intraoperative photos. (A) Intraoperative view of the left neck, superior direction to the right. The sternocleidomastoid muscle is retracted posteriorly with the parotid gland and mandible being retracted anterosuperiorly, maximizing exposure of the lipoma. (B) Postresection image of the specimen. It measured up to 10.6 cm in greatest dimension and consisted of homogenous fibroadipose tissue.

The extent of surgery included a transcervical approach with left levels II‐IV neck exposure as well as parapharyngeal space exploration with facial nerve dissection. We employed facial nerve monitoring throughout the case. Initially, the posterior belly of the digastric and the stylohyoid muscles were divided. This allowed for a wide operative field of exposure of the superior extent of the parapharyngeal space without the need for mandibulotomy, optimizing the dissection and ability to completely excise the mass en bloc (Figure 2). Intraoperatively, the left internal jugular vein was found to be compressed by the mass and diminutive in size, with apparent compensatory hypertrophy of the left external jugular vein. The left facial nerve main trunk, carotid artery, and cranial nerves 10–12 were also identified and all preserved. The digastric was easily reapproximated at the end of the case. Permanent section pathologic analysis revealed unoriented fibroadipose tissue, consistent with a simple lipoma without any concern for sarcomatous changes. The patient’s postoperative course was uneventful. An exam at the 1‐week follow‐up showed intact cranial nerves 10–12, symmetric shoulder strength, and House–Brackmann scores of 1/6 bilaterally. At the 1‐month postoperative visit, the patient’s surgical site was healed appropriately, and he retained full neck and shoulder mobility. There was no evidence of any cranial nerve deficit or dysphagia.

### 2.2. Review of Literature

The first case of PPL was reported in 1977 by Banerjee [12]. A literature search was conducted on PubMed for any subsequently published English‐language case reports or case series including the keywords “parapharyngeal” + “lipoma”. This resulted in the inclusion of 41 articles reporting on PPL and a total of 45 patients (Table 1). Table 1 provides a chronological record of all PPL reports in the literature.

**TABLE 1:** Results of literature search.

Author, year	Patients, n and sex (M/F)	Mean age, years	MRI, *n*	CT, *n*	FNA, *n*	US, *n*	XR, *n*	Max lipoma dimension (cm)	Surgical approach
Loudghiri et al., 2023 [13]	1 M	44	1	1	—	—	—	9.2	Combined cervical and endobuccal approach
Tuan et al., 2023 [14]	1 M	25	1	—	1	1	—	11.0	—
Zhu and Li, 2022 [15]	1 F	2	1	1	—	—	—	—	Combined transoral and transcervical approach
Abdullah et al., 2021 [16]	1 M	4	1	1	1	—	—	6.6	Transcervical
Aslan et al., 2020 [17]	1 M	20	1	—	—	—	—	6.0	Transoral
Lee and Ngai, 2020 [18]	1 F	“Middle‐aged”	1	—	1	1	—	9.2	Transcervical
Raju et al., 2020 [19]	1 M	55	—	1	1	1	—	5.8	Superficial parotidectomy
Marion et al., 2019 [20]	1 M	69	1	—	—	—	—	12.5	Monobloc resection
Hakeem et al., 2018 [21]	1 M	50	1	—	—	—	—	24.0	Transcervical
Singh et al., 2018 [22]	1 F	2	1	—	—	1	—	4.6	Extended submandibular
Baisakhiya et al., 2016 [23]	1 M	45	—	1	1	—	—	9.0	Transparotid
Crowson et al., 2016 [7]	2 M	69	2	1	1	—	—	13.6	1 declined to operate; 1 transcervical
Ahmed et al., 2015 [24]	1 M	9	1	—	—	1	—	7.2	Extended submandibular
Aydin et al., 2015 [25]	1 M	68	1	—	—	—	—	6.0	Transcervical
Garcia‐Ortega et al., 2015 [26]	1 M	48	—	1	1	1	—	16.0	Cervical exploration
Luczak et al., 2015 [27]	1 M	75	—	1	—	—	—	8.5	Lateral cervical
Mendelsohn, 2015 [28]	1 M	56	1	—	—	—	—	5.6	Transoral robotic resection
Pal et al., 2015 [29]	1 F	40	—	1	—	—	—	7.0	Transcervical
Arshad et al., 2013 [30]	1 M	68	1	—	—	—	—	6.6	Transoral robotic resection
Chua et al., 2013 [31]	1 M	53	1	1	—	—	—	4.0	Transoral endoscopic
Casale et al., 2012 [32]	1 M	70	1	—	—	—	—	9.0	Transcervical
Kwon et al., 2010 [33]	1 M	81	—	1	—	—	—	3.0	Transoral pharyngotomy
Rogers et al., 2010 [34]	2 M	29	1	1	—	—	—	6.8, 7.5	2 transcervical
Derin et al., 2009 [35]	1 M	44	—	1	1	—	—	7.0	Parotid exposure through modified Blair incision
Chen, 2006 [36]	1 M	59	1	—	1	—	—	4.0	“Subtotal excision”
Gooskens and Manni, 2006 [37]	2 M, 1 F	49.7	3	—	1	—	—	12.0, 8.0	1 transparotid, 1 total parotidectomy, 1 parotid exploration
McNeill et al., 2006 [38]	1 M	75	1	1	1	—	—	6.0	Declined to operate
Erkan et al., 2004 [39]	1 F	71	1	—	—	—	—	5.5	Transcervical
Singh et al., 2004 [40]	1 F	38	—	1	1	—	—	10.0	Modified Blair incision extending to submandibular
Ulku and Uyar, 2005 [4]	1 M	18	—	1	—	—	—	—	Transcervical
Pellanda et al, 2003 [41]	1 M	53	1	—	—	—	—	9.0	—
Smith et al., 2002 [5]	1 M	49	1	1	—	—	—	—	Transcervical
Baumann et al., 2001 [42]	1 M	45	1	1	—	—	—	7.5	Total parotidectomy and submandiulectomy
Minutoli et al., 2001 [43]	1 F	46	1	1	—	—	—	4.0	“Excision of mass, which was independent of the skull base”
Scott et al., 1999 [6]	1 M	69	—	1	1	—	—	—	Transcervical
Abdullah et al., 1997 [44]	1 M	60	—	1	—	—	—	7.0	Declined to operate
Elango, 1995 [45]	1 F	55	—	1	—	—	—	4.5	Transcervical
Higashi et al., 1992 [46]	1 M	61	—	1	—	—	—	9.0	—
Kakani et al., 1992 [8]	1 F	12	—	1	—	—	1	—	Transparotid
Yamada et al., 1990 [47]	1 M	31	—	1	—	—	—	—	—
Banerjee, 1977 [12]	1 M	48	—	—	—	—	1	12.0	“Approached through the neck”

*Note:* US, ultrasound.

Abbreviations: CT, computed tomography; FNA, fine‐needle aspiration; MRI, magnetic resonance imaging; XR, X‐ray.

There were 35 (78%) male patients and 10 (22%) female patients with a median age of 49.5 years (mean 46.9, SD 22.1). The median maximum dimension of PPLs measured 7.2 cm (mean 8.2, SD 3.9). Workup involved the combined use of at least two diagnostic modalities in slightly over half (*n* = 23, 51%) of patients. The most commonly used imaging modality was MRI (*n* = 28, 62%), closely followed by CT (*n* = 25, 56%). Lipoma was identified using fine‐needle aspiration (FNA) cytology or biopsy in 13 cases (29%) and with ultrasound in 6 cases (13%). Notably, X‐ray was only used as a diagnostic modality in two early cases prior to 2000. In recent years, there has been a noticeable trend favoring MRI as the preferred preoperative imaging choice.

Patients reported a symptom history ranging from 10 weeks to several decades prior to seeking treatment. Lipoma site enlargement or swelling was the most frequent symptom, reported in two‐thirds (*n* = 30, 67%) of patients, and was generally described as a progressively growing painless mass. Dysphagia (*n* = 13, 29%), dysphonia (*n* = 7, 16%), and throat lump, discomfort, fullness, or foreign body sensation (*n* = 8, 18%) were also commonly reported. There were seven (16%) cases associated with obstructive sleep apnea. Additionally, one patient reported radiating neck pain, and there were two reports of hearing disturbance.

Of the 45 patients, 42 (93%) elected to undergo surgical resection of the lipoma. The transcervical surgical approach was the most popular (*n* = 16, 36%), followed by transparotid (*n* = 7, 16%) and transoral (*n* = 5, 11%). Other surgical options included three submandibular approaches and three combined approaches, plus one monobloc resection.

Follow‐up ranged from 1 week to 10 years, and most patients reported complete and immediate resolution of symptoms following lipoma removal. Transiently, six (13%) patients reported postoperative complications, including three cases of minor facial weakness and nerve paralysis which all improved with time. Only two patients experienced major permanent complications. The first patient underwent surgery at an unreported institution and presented with Horner’s syndrome, first‐bite syndrome, left vagus nerve neuropathy, and chronic left‐sided headache to Crowson et al. 15 months later for monitoring [7]. The second patient was the only case of lipoma recurrence. Their second operation was complicated by the presence of scar tissue, and postoperatively the patient reported difficulty swallowing and hoarseness [46].

## 3. Discussion

There have been fewer than 50 reported cases of lipomas in the parapharyngeal space since 1977, making their presentation an exceedingly rare occurrence [12]. In cases such as ours, where there is superior extension of a large 10.6 cm mass, thorough preoperative assessment and nuanced surgical planning are essential.

Preoperative assessment of possible PPLs requires consideration of a multitude of factors including patient presentation, tumor characteristics, and anatomical complexity. In this review, most PPLs occurred in middle‐aged men, unlike other parapharyngeal space tumors which have a more balanced sex distribution [48]. Enlarging neck mass was the most common symptom in these patients, and there was generally an absence of additional systemic symptoms, suggesting a benign etiology. Despite their benign nature, the larger size of PPLs on average requires that special care be taken during surgical planning. In this review, the median maximum dimension of PPLs measured 7.2 cm but was reported as large as 24 cm. In contrast, the reported median dimension of other parapharyngeal space tumors in the literature was 4.0 cm [49].

The parapharyngeal space remains a precarious domain for surgical interventions, housing an array of vital neurovascular structures [1, 2], and imaging is useful for not only ruling out concern for malignancy but also guiding the subsequent surgical approach. MRI and CT scans are often recommended to assess the extent and nature of the mass, especially if there is extension into the foramen transversarium of C1 and C2, although this was not the case for our patient. CT imaging alone was sufficient for our diagnostic and surgical planning purposes following a thorough history and physical exam, and it remains commonly used for PPL workup. The increasing MRI adoption observed in recent years may be attributed to greater accessibility and provider preference. MRI provides enhanced soft‐tissue imaging, including fat suppression and neurovascular delineation, which may be particularly beneficial in cases where there is concerning neurovascular involvement. However, it remains more costly and less accessible compared to CT [50]. Ultrasound may be used initially if CT or MRI are unavailable, and FNA can confirm imaging suspicions prior to definitive surgical resection, but this workup is less commonly performed [50].

Multiple PPL surgical approaches have been documented in the literature, including the most common transcervical approach, followed by transparotid and transoral approaches. The transcervical approach is popular due to minimal facial scarring, shorter operative time, and favorable outcomes [51, 52]. However, several reports have highlighted the difficulty in gaining adequate exposure to the upper neck and skull base vessels during a transcervical approach [1, 2, 5, 18]. To avoid mandibulotomy, our case required parapharyngeal space exploration, considering the size and superior extension of the lipoma to the skull base. Additionally, facial nerve dissection was critical, as it provided the necessary surgical access for an extensive dissection while safeguarding crucial anatomical structures. While some authors recommend a transmandibular approach for enhanced superior exposure [18], this technique comes at the expense of significantly slower functional recovery and the need for a potential tracheotomy, in addition to poorer cosmesis [53]. Mandibulotomy may still be indicated when a transcervical approach alone is not feasible, such as for extremely large tumors, malignant tumors, and those with greater skull base involvement. When possible, our approach, coupled with facial nerve dissection, allows for comprehensive exposure without the need for mandibulotomy, thereby significantly reducing surgical morbidity without compromising complete tumor excision. While our modified approach proved successful, we acknowledge the limitations of a single report, and it is crucial for future endeavors to refine and validate the safety and efficacy of our technique.

## 4. Conclusion

PPL management demands intricate preoperative planning, particularly when the mass extends into challenging anatomical territories. While some cases may demand a mandibulotomy, others, as in our experience, can be managed effectively without such invasive techniques. We demonstrate the successful excision of a large and superiorly extensive PPL, employing a transcervical approach with facial nerve dissection. Advanced preoperative imaging, detailed understanding of anatomy, and a tailored surgical approach are key to management of tumors in the parapharyngeal space.

## Author Contributions

Drafting and editing of manuscript: Amy Wang, Nicholas A. Rossi, and Viran Ranasinghe.

Concept, guidance, and review of manuscript: Viran Ranasinghe.

## Funding

No funding was received for this manuscript.

## Disclosure

The authors declare that there are no additional disclosures to report.

## Consent

The need for approval was waived as information pertaining to the case report has been sufficiently anonymized in accordance to ICMJE guidelines.

## Conflicts of Interest

The authors declare no conflicts of interest.

## Data Availability

All data generated or analyzed during this study are included in this published article and can be made available on request through the corresponding author.
